# Ultrastructural Description of *Sarcocystis* Sp. in Cardiac Muscle of Naturally Infected Alpacas (*Vicugna pacos*)

**Published:** 2019

**Authors:** J. Raúl LUCAS, Manuel BARRIOS-ARPI, José RODRÍGUEZ, Stephanie BALCÁZARNAKAMATSU, Jacquelyne ZARRIA, Gislene NAMIYAMA, Noelia TANIWAKI, Omar GONZALES-VIERA

**Affiliations:** 1. Veterinary Institute of Tropical and High Altitude Research, School of Veterinary Medicine, National University of San Marcos, Junin, Peru; 2. Physiology Laboratory, School of Veterinary Medicine, National University of San Marcos, Lima, Peru; 3. Specialized Equipment Laboratory, Faculty of Biological Sciences, National University of San Marcos, Lima, Peru; 4. Electronic Microscopy Nucleus, Adolfo Lutz Institute, Sao Paulo, Brazil; 5. Department of Pathology, Microbiology and Immunology, School of Veterinary Medicine, University of California, Davis, CA, USA

**Keywords:** Alpacas, Apicomplexa, *Sarcocystis*, Transmission electron microscopy, Heart

## Abstract

**Background:**

Recently, it was proposed the name of *Sarcocystis masoni* n. sp. for the *Sarcocystis* that causes microcyst in skeletal muscle of South American camelids. However, there are no ultrastructural reports of microcysts of *Sarcocystis* in cardiac muscle of alpacas. This study reports ultrastructural features of microcysts of *Sarcocystis* sp. from cardiac muscle of naturally infected alpacas.

**Methods:**

Thirty alpacas (age range: three to five years) from the province of Junin, Peruvian Central Andes, were included in this study in January 2015. Cardiac muscle samples were evaluated by histology and transmission electron microscopy.

**Results:**

Bradyzoites in cysts had typical characteristics of Apicomplexa including organelles, a large nucleus, micronemes, dense bodies, and polysaccharide granules. Moreover, cysts had a thin wall with numerous, short, finger-like shapes with rounded tip protrusions (0.51 × 0.17 μm).

**Conclusion:**

*Sarcocystis* sp. from the heart and *S. masoni* n. sp. from the skeletal muscle have similar ultrastructural characteristics.

## Introduction

*Sarcocystis* spp. cause sarcocystosis in humans and animals worldwide. Two zoonotic species, *S. suihominis* and *S. hominis*, cause the parasitic food-borne disease sarcocystosis and are both acquired by the consumption of undercooked meat. Furthermore, sarcocystosis negatively affects food safety that results in economic losses by producing condemnation in slaughterhouses (1, 2).

In the high Andean region of Peru, South American camelids (SAC) are an important economic resource. The alpaca is a SAC that acts as an intermediate host of the two recognized *Sarcocystis* spp. affecting South American camelids. *S. aucheniae* develops macrocysts in striated skeletal muscles while *S.* sp. (well-known as a *S*. “*lamacanis*”) produces microcysts mainly in cardiac muscle and there are biological and genetic differences between them (3–6). Recently, Moré et al (7) considered *Sarcocystis* sp. *“lamacanis”* as *nomen nudum*, proposing the name *S. masoni* for *Sarcocystis* sp. forming microcysts in skeletal muscle of SAC.

Ultrastructural characterization of the primary cyst wall (CW) is considered the most important criteria in differentiating *Sarcocystis* spp. (8, 9). Despite this, there are scarce ultrastructural descriptions of *Sarcocystis* from alpacas, and there is no information confirming that *Sarcocystis* sp. “*lamacanis*” infecting cardiac muscle and *S. masoni* n. sp. in skeletal muscle are the same species.

Therefore, the aim of this study was to report the ultrastructural features of *Sarcocytis* sp. from cardiac muscle of naturally infected alpaca of the Peruvian Andes.

## Materials and Methods

Samples of cardiac muscle were obtained from thirty alpacas (age range: three to five years) from the province of Junin in the Central Andean region of Peru 3,300 meters above sea level in January 2015. Samples of cardiac muscle (2 cm^2^) per animal were obtained from the Ninancaca slaughterhouse (Junin). Half of each sample was fixed in 10% buffered formalin for histological analysis, and the other half was placed in 2% glutaraldehyde in Sorensen's phosphate buffer 0.1 M (pH 7.2) for 24 h as the first step for transmission electron microscopy (TEM) analysis.

For histology, samples were processed following the standard hematoxylin and eosin staining procedures in the Laboratory of Animal Pathology of the National Service of Animal Health, Peru. Samples were screened for *Sarcocystis* spp. microcysts under low magnification with a light microscope. Heart tissues containing microcysts were submitted for TEM analysis to the Electron Microscopy Department at the Adolfo Lutz Institute, Brazil.

Fragments of fixed cardiac muscle were fixed in 1% Osmium Tetroxide, dehydrated in acetone series, and embedded in Epoxy resin. Semithin sections were stained with 1% metilene blue and examined by light microscopy at high power magnification to select areas of muscle containing cysts.

Ultrathin sections were obtained in a Sorvall Ultramicrotome, stained with uranyl acetate and lead citrate and observed under a JEOL JEM1011 Transmission Electron Microscopy operating at 80kV. Images were recorded with a Gatan 785 ES 1000W Erlangshen camera.

## Results

### Light Microscopy description

**Fig. 1:**
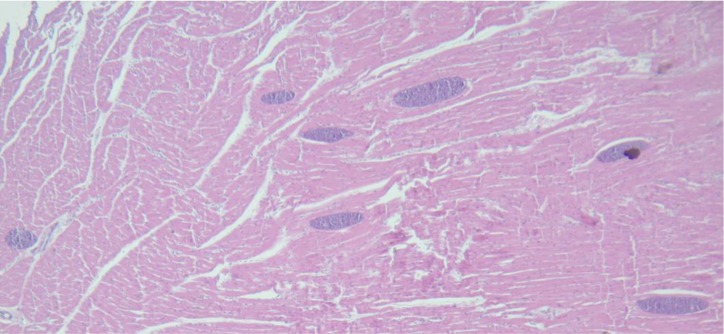
Cardiac muscle, alpaca. Microscopy of cardiac fibers muscle containing microcysts of *Sarcocystis* sp. *“lamacanis”*. HE. 100X

All the 30 samples of alpaca cardiac muscle contained microcysts of *Sarcocystis* sp. “*lamacanis*”. Microcysts were high in number (Fig. 1), but no inflammatory reaction was observed against the microcysts. All microcysts were similar in size, with a mean of 43.43 μm (range: 22.67–74.18 μm) in width, and 78.24 μm (range: 28.05–280.82 μm) in length.

### Ultrastructural description

**Fig. 2:**
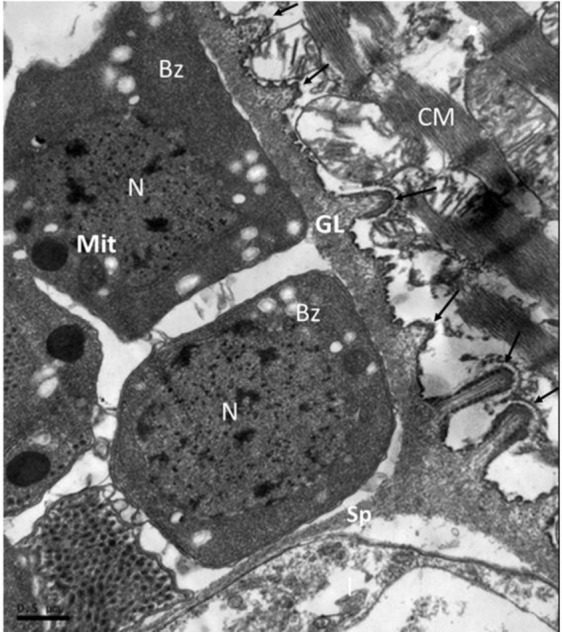
Cardiac muscle, alpaca. Longitudinal section of *Sarcocystis* sp *“lamacanis”.* Note the ground substance layer (GL) separating bradizoytes (Bz) by septas (Sp). The cyst wall is characterized for presenting short finger-like villi protrusions (VP) (arrow black) of different sizes and with rounded tips. GL: Granular layer, CW: Cyst wall, N: Nucleus; Mit: mitochondria; Sp: Septa; CM: Cardiac muscle fibers. TEM. Bar = 0.5 μm

**Fig. 3:**
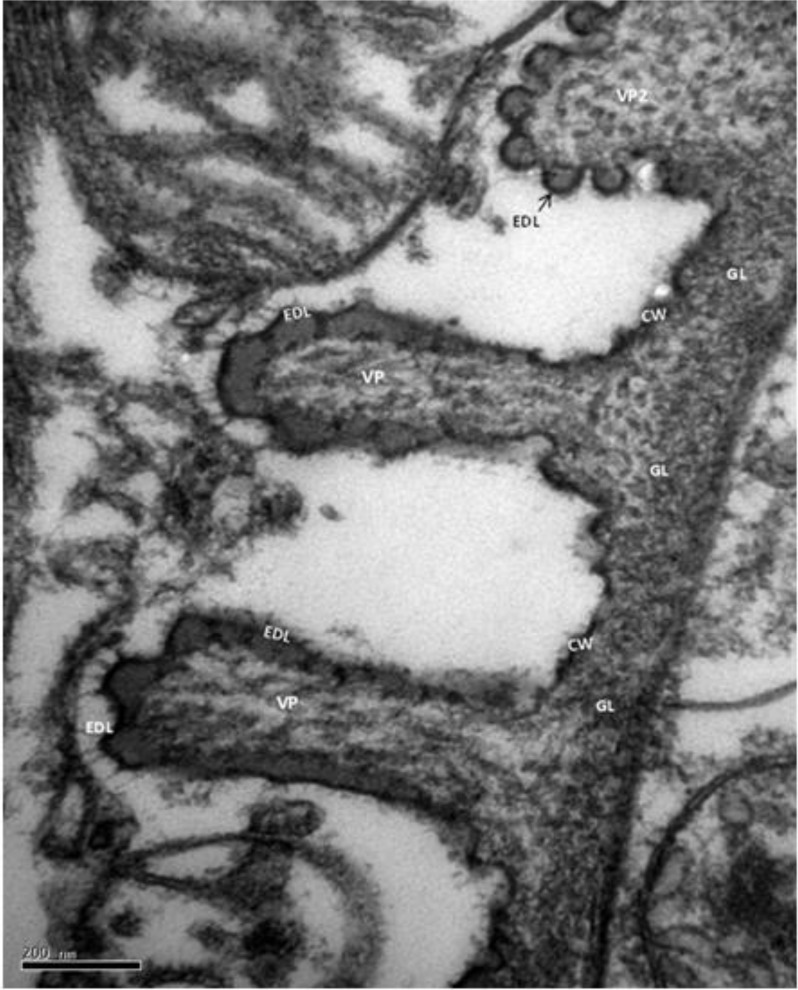
Observe three villus protrusions (VP) of Sarcocystis. Note electron dense layer (EDL) of varying thickness lining the villus protrusions. VP occasionally showed a base a bit wider than the tip (VP2). GL: Granular layer, CW: Cyst wall. TEM. Bar = 200 nm

**Fig. 4:**
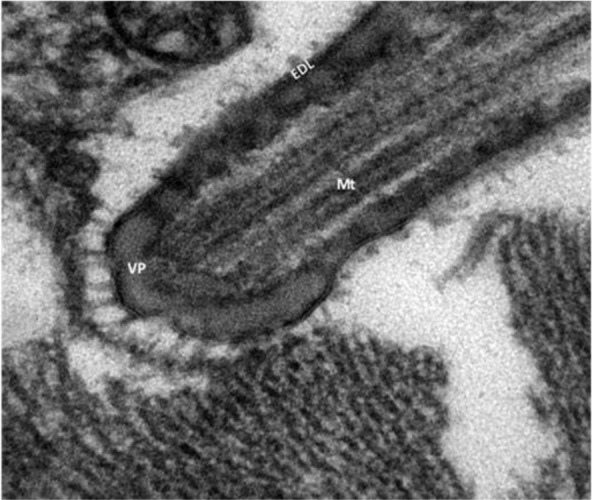
Cardiac muscle, alpaca. High magnification of a villus protrusion (VP), showing microtubules (Mt). “Hobnailed” appearance of electron dense layer (EDL) is evident. TEM. Bar = 100 nm

**Fig. 5:**
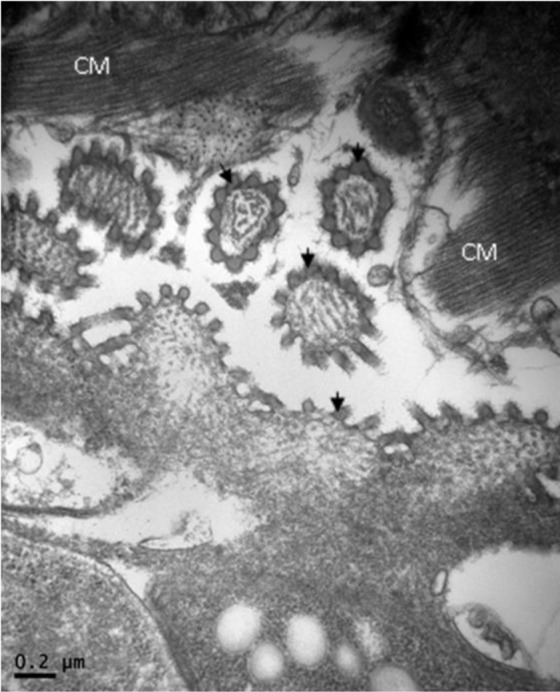
Cardiac muscle, alpaca. Transversal section of the cyst wall of *Sarcocystis* sp. “*lamacanis*”. Note the presence of “Hobnailed” appearance of electron dense layer (EDL)-like vesicles (arrows). CM: Cardiac muscle fibers. TEM. Bar= 0.2 μm

All of the microcysts were bounded by thin primary cyst walls (CW) having a mean of 33.9 nm (range: 3.1–74.2 nm) in thickness (Fig. 2). At the surface (Fig. 2), the CW formed tubular, short, villi projections or protrusions (VP) that were not seen in sections stained with hematoxylin and eosin (Fig. 1). The VP had a finger-like shape with rounded tips and were a mean of 0.51 μm (range: 0.16–1.06 μm) long and 0.17 μm (range: 0.04–0.56 μm) wide. The VP occasionally showed a mean of 0.29 μm base (range: 0.14–0.56μm) that was slightly wider than the mean 0.12 μm tip (range: 0.07–0.21μm) (Fig. 3). The VP were spaced a mean of 0.46 μm (range: 0.23–1.44 μm) apart, had longitudinally arranged microtubules and hair-like projections on the VP tip (Fig. 4) and an external electron dense layer, which seems to be vesicles in transverse section (Fig. 5).

**Fig. 6:**
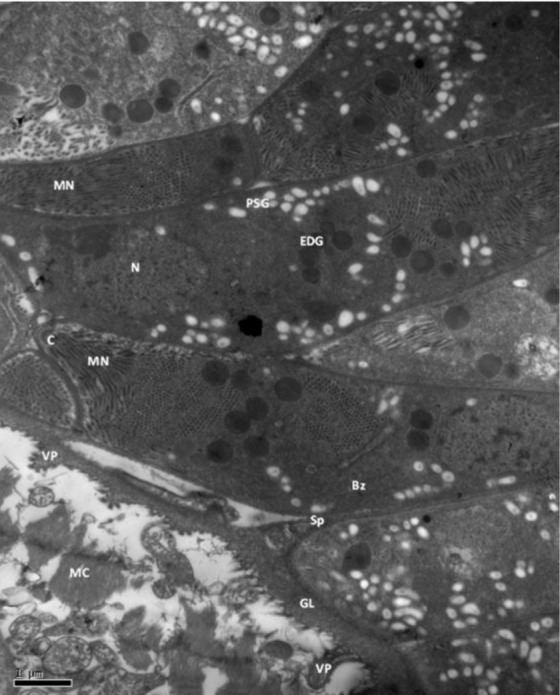
Cardiac muscle, alpaca. Bradyzoite (Bz) contains polysaccharide granules (PSG), nucleus (N), endoplasmic reticulum (ER), Golgi apparatus (GA), electron dense granules (EDG), micro-nemes (MN). Pl: Plasmaleme, Im: Inner membrane complex, Sp: Septa. TEM. Bar = 0.5 μm

Bradyzoites (BZ) displayed a uniformly thick ground substance layer (GL), 236.3 nm (range: 99.4–464 nm) thick, immediately beneath the CW and at the bottom of the protrusions (Fig. 2). The GL extended to the interior of the cysts and formed septa (SP) that subdivided the cysts into many compartments, enclosing numerous BZ (Fig. 6). All of the examined sarcocysts were mature as they contained many BZ (1.95–3.53 × 2.69–10.68 μm), which showed typical characteristics of other *Sarcocystis* spp., i.e. single membrane (24.47–31.72 nm thick), completely developed organelles (i.e., Rugose endoplasmic reticule, microtubules, mitochondria), a large nucleus, micronemes, and numerous polysaccharide and electron dense granules in the central and caudal region (Fig. 6).

## Discussion

All the samples were positive for *Sarcocystis* sp. “lamacanis”, which was expected because the sarcocystosis is a hyperendemic disease for SAC of the Andean region. Moreover, our results agree with other studies that reported frequencies from 87% to 100% (2). The in-grained practices related to the lack of knowledge of parasites transmission could favor sarcocystosis dissemination in Peruvian Highlands. For instance, Andean farmers feed their shepherd dogs with infected tissues during home slaughtered of SAC (3).

All sarcocystis species possess a wall that often has unique ultrastructural characteristics that are used to distinguish *Sarcocystis* spp. within the same intermediate host (10–15). This is particularly useful to identify zoonotic and non-zoonotic *Sarcocystis* spp. species in cattle (15).

The present study reports a TEM description of *S.* sp. from alpaca cardiac muscle and our results show that the CW of *Sarcocystis* sp. has finger-like rounded tip protrusions. We depicted that *Sarcocystis* sp. from heart of alpacas has similar ultrastructural characteristics of CW to those of *S. cameli*, parasite of camels classified earlier (16) as “type 9j.”

Moreover, these characteristics are similar to those previously reported for *S. masoni* in the skeletal muscle of camelids (7). However, the microcysts and VP of *S. masoni* (7) were longer than in our description. Apparently, the microcysts of *Sarcocystis* sp “lamacanis” are shorter than those from skeletal muscle because cardiac muscle is a denser and less elastic tissue which could restrict microcysts growth. Similarly, microcysts of *S. gigantea* could had a different shape and size depending of the tissue they are found (17). Other parasitic cysts showed similar development restriction within dense and inelastic tissues, i.e. hydatid cyst in hepatic tissue (18).

The cyst wall of *S. aucheniae* has numerous cauliflower-like protrusions that are densely distributed (3, 7). Therefore, it is confirmed that the *Sarcocystis* sp. producing microcysts in cardiac muscle of camels is a different species from the *S. aucheniae*. TEM also permits us to establish that the organelles of *Sarcocystis* sp. from heart show similar characteristics with other *Sarcocystis* spp.

## Conclusion

This ultrastructural description of CW confirms that *S.* sp. affecting cardiac muscle of alpacas and *S. masoni* n. sp. reported from skeletal muscle of SAC are similar. These results increase the biological knowledge of *Sarcocystis* spp. in SAC.
